# Nasal Sublesional Bevacizumab Injections as Adjuvant Treatment for Diffuse Sinonasal Exophytic Papillomas

**DOI:** 10.3390/jcm15020723

**Published:** 2026-01-15

**Authors:** Anna Penella, Adriana Michavila, Marta Fulla, Elisabet Leiva Badosa, Aina Brunet, Maria Foglia-Fernández, Xavier González-Compta

**Affiliations:** 1 Otorhinolaryngology Department, Hospital Universitari de Bellvitge, Universitat de Barcelona (UB), Feixa Llarga, s/n, 08907 L’Hospitalet de Llobregat, Spain; apenella@bellvitgehospital.cat (A.P.); adrianamichavila@gmail.com (A.M.); mfulla@bellvitgehospital.cat (M.F.); abrunet@bellvitgehospital.cat (A.B.); mariafoglia@bellvitgehospital.cat (M.F.-F.); 2 Departament de Ciències Clíniques, Facultat de Medicina i Ciències de la Salut, Universitat de Barcelona (UB), Feixa Llarga s/n, 08907 L’Hospitalet de Llobregat, Spain; 3 Institut d’Investigació Biomèdica de Bellvitge (IDIBELL), 08908 L’Hospitalet de Llobregat, Spain; eleiva@bellvitgehospital.cat; 4 Pharmacy Department, Hospital Universitari de Bellvitge, Feixa Llarga, s/n, 08907 L’Hospitalet de Llobregat, Spain

**Keywords:** adjuvant, bevacizumab, diffuse, exophytic papilloma, sinonasal

## Abstract

**Background/Objectives:** Diffuse sinonasal exophytic papillomas (DSNEPs) are rare entities, with similarities to recurrent respiratory papillomatosis (RRPs). DSNEP treatment is usually based on surgical excision, but the recurrence rate is high. Bevacizumab injections have been increasingly used as an adjuvant option for RRP, but their role in DSNEP treatment remains unknown. The current study describes the preliminary experience, safety profile, and exploratory outcomes of sublesional bevacizumab injections following surgical excision. **Methods:** We undertook a retrospective, single-centre study of a cohort of patients diagnosed with DSNEP between 2011 and 2018. All patients were treated with surgical excision and sublesional bevacizumab injections. The effect of bevacizumab was evaluated using a severity score developed to quantify lesion size and the extent of affected areas in each patient. **Results:** Seven patients diagnosed with DSNEP were treated. All patients were male, with a median age at diagnosis of 42 years [38–44.5]. Human papillomavirus (HPV) DNA was detected in all patients: HPV-11 in six cases (85.7%) and HPV-6 in one case (14.3%). Bevacizumab was injected into the submucosa of their surgical sites. The median follow-up was 55.5 months [40.85–82.73]. Most patients (85.72%) presented recurrence, with a median of 3 years [1.5–4]. A statistically significant reduction in the severity score was observed (*p* = 0.017), although this finding cannot be attributed solely to bevacizumab due to study design limitations. No relevant complications were reported. **Conclusions:** Nasal sublesional bevacizumab injections were well tolerated and feasible as an adjuvant approach to DSNEP. Larger prospective studies are needed to confirm its safety and assess its potential benefit.

## 1. Introduction

**Figure 1:**
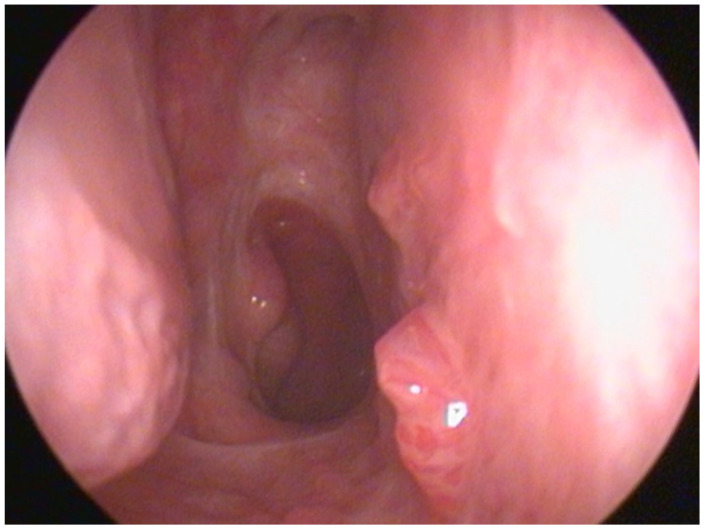
Papillomatous lesions located in septum and floor of right nasal cavity.

Sinonasal exophytic papillomas (SNEPs) can be found as focal lesions or as a multifocal/diffuse nasal pathology (Figure 1) known as diffuse sinonasal exophytic papillomas (DSNEPs) [1]. The light microscopic appearance of these diffuse sinonasal papillomas (DSNEPs) is analogous to squamous cell papillomas in other mucosal sites, like the larynx, characterised by branching fronds of mucosa covered by stratified squamous epithelium and supported by a connective tissue (fibrovascular) scaffold [2,3]. DSNEPs have been described to have similarities to recurrent respiratory papillomatosis (RRP), as both are benign human papillomavirus (HPV)-related lesions of the upper aero-digestive tract with a tendency toward multiple recurrences [1].

Recent work from our own group further characterised the histological and HPV-related features of this DSNEP cohort in comparison with focal SNEP, highlighting its younger age at presentation, predominant septal involvement, high recurrence rate, and strong association with HPV-11 [1]. Building on those findings, the present study evaluated the clinical response and safety of sublesional bevacizumab injections as a potential adjuvant approach to DSNEP.

Regarding DSNEP treatment, management is typically based on endoscopic sinonasal excision of these lesions using a microdebrider or laser photocoagulation [2]. The close anatomical relationship between the nasal cavity, orbit, and skull base underscores the need for meticulous surgical planning, as highlighted by Raponi et al. [4]. Despite appropriate surgical management, recurrence rates remain high, ranging from 22–36% [2].

Local injection of cidofovir has been recently studied with favourable results as an adjuvant treatment for DSNEP [5], as has topical interferon alpha-2b treatment in focal sinonasal exophytic papillomas (FSNEPs) [6], but to our knowledge, sublesional bevacizumab injection has not been previously evaluated regarding SNEP.

Bevacizumab is a recombinant monoclonal humanised immunoglobulin G1 antibody that inhibits vascular endothelial growth factor A and thus blocks angiogenesis. Local injection of bevacizumab in larynx papillomas was first described in 2009 by Zeitels et al. [7]. The same group published several papers confirming the safety and efficacy of this treatment between 2009 and 2012 [7,8,9]. During the same period, several patients with uncontrolled DSNEP were diagnosed at our centre, prompting us to explore sublesional nasal bevacizumab injections under the rationale that DSNEP may represent the nasal expression of RRP. Furthermore, nasal submucosal injection of bevacizumab was described to be a safe treatment in patients with hereditary haemorrhagic telangiectasia in 2012 [10]; over the years, it has been proven to be safe and effective in preventing recurrences in laryngeal respiratory papillomatosis with sublesional injections [11,12]. In addition, a recent RRP study proposed an image-based quantitative method to assess bevacizumab response, showing strong concordance with the Derkay score and further supporting the drug’s safety and clinical effectiveness [13].

The existing body of literature on DSNEP remains limited, and no published studies have evaluated the feasibility, safety, or potential benefit of sublesional bevacizumab injections, or its management. Given the rarity of this condition and the absence of prior data on local bevacizumab administration for DSNEP, even preliminary observational findings may provide valuable guidance for future research. In this context, the aim of the present study was to provide an exploratory description of the clinical response and safety profile of sublesional bevacizumab injections administered as an adjuvant approach to patients with DSNEP.

## 2. Materials and Methods

This study was an observational retrospective single-centre analysis of all consecutive patients diagnosed with DSNEP in a tertiary centre from 2011 to 2018, with a follow-up of at least 32 months. DSNEP was defined as nasal papillomatous multifocal lesions diagnosed by nasal endoscopy and confirmed by biopsy. The histopathological and HPV analyses of this cohort were previously reported in a comparative study [1], although the present work represents an independent clinical evaluation focused on the effect of bevacizumab treatment.

Inclusion criteria were patients with a DSNEP diagnosis and age ≥ 18 years old.

All patients were offered sublesional bevacizumab injections as an off-label treatment for their condition, under the rationale that DSNEP constitutes the nasal expression of RRP, for which this therapy was already in clinical use. Written informed consent was obtained from all patients prior to the administration of bevacizumab. All patients were treated with surgical excision and sublesional bevacizumab injections (an injection was performed during each surgery). Bevacizumab was prepared in a syringe under aseptic conditions at a concentration of 12.5 mg/3 mL at the Pharmacy Department. Dosing variability among patients reflected differences in lesion extension; however, the absence of a predefined dosing schedule represents a limitation affecting reproducibility.

The following data were recorded:

- Demographics
- Related to HPV: history of known HPV infection, presence of papillomatous lesions in other areas of the respiratory tract, HPV vaccination status, HPV genotype
- Related to DSNEP: symptoms, localization in the nasal cavity
- Previous treatments related to DSNEP
- Number of recurrences and time to recurrence after treatment with bevacizumab
- Related to bevacizumab injections: number of injections administered, the concentration and cumulative dose used, complications after injections

The effect of injected bevacizumab on DSNEP was evaluated by a severity score to assess the sizes of lesions and areas affected in each case, taking as a reference the RRP score by Derkay [14]. According to the size of the lesion, scores were 0: no lesion, 1: superficial lesion, 2: raised lesion, and 3: bulky lesion. Data were collected from different regions of the sinonasal cavity (bilaterally) to assess disease extension as follows: the vestibule, paranasal sinuses, anterior septum, posterior septum, inferior turbinate, lateral wall (including meatus and superior and middle turbinates), floor of the nasal cavity, roof of the nasal cavity, soft palate, and cavum. The maximum score value was 60 points.

Clinical stability of the lesions was defined as the absence of any increase in size or number of lesions over time. Remission was considered when no new lesions appeared during the follow-up period. Surgical intervals were not systematically analysed, as operating room availability and clinical workload at our centre did not allow these intervals to be used as a reliable outcome measure; however, severity scores after each injection were recorded when available to better describe disease evolution between procedures.

Categorical variables were described using the number of observations and percentages. Continuous variables were described using the median and interquartile range (IQR). A Wilcoxon signed-rank test was conducted to compare DSNEP severity scores before and after bevacizumab injections. Data management and statistical analyses were performed using STATA/BE17.0.

This study was approved by the Institutional Ethics Committee (PR413/20).

## 3. Results

Seven patients diagnosed with DSNEP were treated at our centre between 2011 and 2018 (Table 1). All patients were male, with a median age at diagnosis of 42 years [38–44.5]. Of these, three patients (42.9%) were referred with an initial diagnosis of DSNEP. Two patients (28.6%) presented with recurrent lesions following previous DSNEP surgery. One patient (14.3%) had initially been misdiagnosed with persistent inverted papilloma, and the correct DSNEP diagnosis was established at our centre. Another patient (14.3%) presented with mixed DSNEP (inverted and exophytic) and had undergone five prior surgeries, including two with cidofovir injections.

Four patients (57.1%) presented unilateral disease, three of them in the right fossa, while the other three (42.9%) had lesions in both fossae.

HPV-DNA was detected in all patients: HPV-11 in six cases (85.7%) and HPV-6 in one case (14.3%). None of these patients had a history of laryngeal papillomatosis.

Bevacizumab was injected into the submucosa of surgical sites after the resection of papillomas with a microdebrider or vaporization with CO_2_ laser. Bevacizumab 12.5 mg/3 mL was injected during every surgery at volumes ranging from 1.25 to 6 mL. Most patients (85.7%) presented with recurrences, with a median of three recurrences [1.5–4].

A statistically significant reduction in severity score was observed before and after bevacizumab injections (*p* = 0.017) (Table 2); however, this reduction cannot be interpreted as a direct effect of bevacizumab due to concurrent surgical intervention and the lack of a comparator group. Four patients achieved clinical stability after one to four procedures, with new lesions that appeared after the injections, but remained stable. One patient presented complete remission after one injection. One patient could not be examined after the fourth surgery with bevacizumab injection because of unrelated death 2 weeks after surgery, but after the third injection he showed a reduction in the severity score.

Complications after surgical excision and bevacizumab injection were reported only in one patient who presented with septal perforation, synechiae, and soft palatal dehiscence. In six patients, no side effects after bevacizumab injection were reported.

A comparison between our study and the findings of Chatelet et al. on intralesional cidofovir treatment for DSNEP is presented in Table 3.

## 4. Discussion

DSNEPs are rare entities. Only a few cases have been reported in the literature [1,2,5], and surgical treatment has a high rate of recurrence [2]. Adjuvant therapies may therefore represent an attractive option, as has been described in other HPV-related papillomatous diseases such as laryngeal RRP. Chatelet et al. [5] described favourable outcomes following intralesional cidofovir injection for the treatment of multifocal sinonasal exophytic papillomatosis. More recently, Martínez-Calvo et al. [15] reported a case of sinonasal papillomatosis treated with intravenous bevacizumab with significant improvement in the lesions. However, to the best of our knowledge, no previous studies have specifically evaluated the feasibility and clinical effects of sublesional bevacizumab injections on DSNEP.

The nasal involvement of RRP has been previously described, but until recently, it has never been characterised [1]. DSNEP can be considered the nasal expression of RRP, as both conditions share a diffuse, multifocal, and recurrent behaviour and are related to HPV infection of the upper aerodigestive tract. Injected bevacizumab has been shown to improve disease control in laryngeal RRP [9,11,12]; however, extrapolation to DSNEP should be approached cautiously given anatomical differences and the lack of controlled data in this setting. Based on these considerations and the occurrence of several recurrent DSNEP cases in our centre between 2011 and 2018, the use of sublesional bevacizumab injections was explored as an adjuvant approach. In this exploratory article, we describe the clinical outcomes and safety profile of adjuvant sublesional bevacizumab injections in patients with DSNEP. Due to its low prevalence, a small cohort was collected. Nevertheless, the demographic characteristics of our cohort are consistent with previously published series [2,5,16].

In our study population, the predominant HPV genotype was HPV-11 (85.7%). Similar results were published by Chatelet et al. [5] and Glâtre et al. [2], with HPV-11 genotype prevalences of 83.3% and 92%, respectively. In those studies where DSNEP and FSNEP were not separately analysed [16], the HPV-11 genotype prevalence was lower (50%).

In a systematic review of bevacizumab for laryngeal RRP treatment published by Pogoda et al. [17], they concluded that in 62% of their cases a prolongation of the post-bevacizumab surgical interval was achieved compared with inter-surgical intervals prior to injection. Although prolongation of the surgical interval has been proposed as a marker of clinical benefit in RRP, in our study surgical interval times could not be systematically analysed. However, a statistically significant reduction in DSNEP severity scores was observed after bevacizumab injections (Table 2). This finding should be interpreted with caution, as surgery and bevacizumab were administered concurrently and no comparator group was available.

In our cohort, 85.7% of patients experienced recurrence despite receiving adjuvant bevacizumab after surgery, highlighting the aggressive behaviour of DSNEP. This rate is higher than the 33% recurrence reported by Chatelet et al. [5] after intralesional cidofovir treatment. In both studies, HPV-11 was the predominant genotype and the median age of patients was similar. However, cidofovir injection preceded surgical resection, while bevacizumab injection followed the excision of lesions, and the number of surgeries before injections was significantly higher in Chatelet et al. [5] compared with our study (Table 3). In a recent systematic review and indirect meta-analysis (Zagzoog et al.) [11] comparing cidofovir and bevacizumab intralesional injection in laryngeal RRP, both treatments showed similar results, and bevacizumab was found to show significantly higher partial remission. Taken together, these findings suggest that both treatments may have comparable effects on DSNEP. The differences observed between our study and that of Chatelet et al. [5] were therefore likely influenced by small sample sizes, differences in treatment timing, and other confounding factors, including the intrinsic aggressiveness of the disease.

Other adjuvant therapies, like topical interferon alpha-2b, have been studied for treating FSNEP, but not DSNEP. Inga et al. [6] studied the effect of topical interferon alpha-2b after surgery as an adjuvant treatment for FSNEP. They conducted a study with 78 patients divided in two groups, receiving either topical interferon alpha-2b after surgery or surgery alone as the control group. Recurrence at the 3-month follow-up showed no significant differences, in contrast to the 6-month follow-up, when the treated group exhibited a significantly lower recurrence rate compared to the control group (39.5% vs. 12.5%, respectively). Topical interferon alpha-2b could also be an interesting option for treating DSNEP, but further studies should be performed in order to establish its effectiveness and safety.

The intralesional concentration of bevacizumab in this study was the same as that used for laryngeal lesions. However, the injection volume was larger considering the larger extension of lesions in the nasal fossae compared with those in the larynx. The dosing schedule consisted of sublesional injection of bevacizumab after lesion excision, analogous to laryngeal lesions treatment at our centre. Although damage in the mucosa induced by surgery can diminish bevacizumab submucosal concentration, we believe that bevacizumab injection before lesion excision could imply a major loss of therapeutic concentration. Likewise, we consider that isolated bevacizumab injection without papilloma resection would be an insufficient measure when treating such extended lesions. The sequelae in our cohort (one case of septal perforation, nasal synechiae, and palatal dehiscence) were considered secondary to the surgical procedure itself rather than to bevacizumab injection, consistent with findings reported in a recent systematic review [18]. The palatal surgery was relatively aggressive, and the patient presented with extensive papillomatous lesions, which required wide mucosal resections that may have been directly related to the observed sequelae.

Taken together, these findings suggest that the combination of surgical excision and sublesional bevacizumab injections is feasible and well tolerated, and that no complications appeared was attributable to bevacizumab itself. However, given the study design, these results cannot be interpreted as evidence of treatment efficacy. Nevertheless, reductions in severity scores suggest a potential, but unproven, therapeutic signal that warrants further investigation.

The results of this study must be considered in the context of some limitations. The main limitations include the retrospective design, small sample size, and absence of a control group treated with surgery alone. In addition, the concurrent administration of surgery and bevacizumab precludes isolating the independent contribution of the drug. Although a statistically significant reduction in severity score was observed, this finding must be interpreted with caution, as bevacizumab was administered immediately after surgical debulking and no comparator group was available. Consequently, the clinical improvement observed likely reflects the combined effect of surgery and adjuvant therapy rather than the isolated effect of bevacizumab.

Further limitations include those inherent to the cohort study design, as well as variability in injection volume and the absence of a predefined dosing schedule, which affect reproducibility and highlight the need for standardized protocols. Surgical interval analysis could not be performed as a reliable outcome measure due to clinical workload and limited operating room availability at our centre. Furthermore, systematic endoscopic evaluations between surgeries were not available, and no additional intermediate assessments could be retrieved. The effective submucosal concentration of bevacizumab after injection also remains unknown. Considering the low incidence and prevalence of DSNEP, future prospective, multicentre studies with standardized protocols are needed to better define the role of adjuvant therapies for this condition.

## 5. Conclusions

In conclusion, DSNEP may be classified as an uncommon condition and can be considered the nasal expression of RRP. Surgical treatment usually fails to achieve proper control of lesions.

Sublesional bevacizumab injections after surgical excision appear safe and feasible for treating DSNEP. Although reductions in severity scores were significant, these findings cannot establish efficacy due to study design constraints. Larger, prospective, multicentre studies with standardized dosing and control groups are required before bevacizumab can be considered an evidence-based adjuvant therapy for DSNEP.

## Figures and Tables

**Table 1:** Clinical characteristics of patients, side effects, and follow up.

Patient No.	Sex	Age at Diagnosis	HPV Vaccination	HPV Genotype	Number of Surgeries Prior to Bevacizumab Injection	Number of Recurrences After Bevacizumab Injection	Side Effects	Number of Months of Follow-Up	Status at the End of Follow-Up
1	M	46	Yes	11	5	4	None	163	Stable lesions
2	M	30	Yes	11	0	10	Septal perforation, synechiae, and palatal dehiscence	103	Stable lesions
3	M	43	No	6	0	1	None	55	Stable lesions
4	M	42	Yes	11	1	2	None	48	Stable lesions
5	M	62	No	11	1	3	None	32; Exitus 2 weeks after surgery due to unrelated cause	NA *
6	M	39	No	11	1	4	None	59	Stable lesions
7	M	37	No	11	0	0	None	32	No lesions

* NA: Not available.

**Table 2:** Outcomes before and after bevacizumab sublesional injections. (**A**) Descriptive data. (**B**) Wilcoxon signed-rank test comparing diffuse sinonasal exophytic papilloma (DSNEP) severity scores before and after bevacizumab injections.

(A)											
	DSNEP Severity Score										
Patient Nº	Before Bevacizumab	After 1st Injection	After 2nd Injection	After 3rd Injection	After 4th Injection	After 5th Injection	After 6th Injection	After 7th Injection	After 8th Injection	After 9th Injection	After Last Injection (Last Follow Up)
1	8	4	9	12							2
2	13	3	8	9	4	15	14	12	6	12	7
3	8										1
4	4	6									1
5	8	6	2								5
6	6	4	4	5							5
7	6										0
**(B)**											
**Severity Score (Median, IQR) Before Injections**				**Severity Score (Median, IQR) After Last Injection**				**W**		***p*** **-Value**	
8 [6–8]				2 [1–5]				28		0.017	

**Table 3:** Comparison of our study results with published outcomes of intralesional cidofovir treatment in diffuse sinonasal exophytic papilloma (DSNEP) patients.

Publication	Procedure	Number of Patients	Age (Median, IQR)	Sex	HPV Genotype	Number of Surgeries Before Injection (Median, IQR)	Recurrence	Clinical Improvement
Chatelet et al. [5]	Injected cidofovir before surgical excision of the lesions	6	44 [37.25–55.25]	66% male 33% female	83% HPV 11 17% NA *	3 [1–11.25]	33%	100%
Current study	Injected bevacizumab after surgical excision of the lesions	7	42 [38–44.5]	100% male	86% HPV 11 14% HPV 6	1 [0–1]	86%	100%

* NA: Not available.

## Data Availability

The original contributions presented in this study are included in the article. Further inquiries can be directed to the corresponding author.

## References

[1] Fulla M., Quiros B., Clavero O., Gomà M., de Andrés-Pablo Á., Pavon M.À., Penella A., Alemany L., González-Compta X., Mena M. (2024). Clinical, Histological, and HPV-Related Factors Associated to Diffuse Presentation of Exophytic Nasal Papillomas. J. Clin. Med..

[2] Glâtre R., De Kermadec H., Alsamad I.A., Badoual C., Gauthier A., Brugel L., Parra C., Coste A., Prulière-Escabasse V., Bequignon E. (2018). Exophytic sinonasal papillomas and nasal florid papillomatosis: A retrospective study. Head Neck.

[3] Syrjänen S., Syrjänen K. (2021). Hpv-associated benign squamous cell papillomas in the upper aero-digestive tract and their malignant potential. Viruses.

[4] Raponi I., Giovannetti F., Buracchi M., Priore P., Battisti A., Scagnet M., Genitori L., Valentini V. (2021). Management of Orbital and Brain Complications of Sinusitis: A Practical Algorithm. J. Cranio-Maxillofac. Surg..

[5] Chatelet F., Vinciguerra A., Marc M., Herman P., Verillaud B. (2024). Intralesional cidofovir injections for the treatment of multifocal exophytic sinonasal papilloma. Int. Forum Allergy Rhinol..

[6] Inga P., Pavel T., Tatiana D., Svetlana S., Timur S., Irina A., Andrey B., Vladimir P., Anastasia K., Irada I. (2024). Interferon alpha-2b treatment for exophytic nasal papillomas and human papillomavirus infection. Braz. J. Otorhinolaryngol..

[7] Zeitels S.M., Lopez-Guerra G., Burns J.A., Lutch M., Friedman A.M., Hillman R.E. (2009). Microlaryngoscopic and Office-Based Injection of Bevacizumab (Avastin) to Enhance 532-nm Pulsed KTP Laser Treatment of Glottal Papillomatosis. Ann. Otol. Rhinol. Laryngol..

[8] Best S.R., Friedman A.D., Landau-Zemer T., Barbu A.M., Burns J.A., Freeman M.W., Halvorsen Y.D., Hillman R.E., Zeitels S.M. (2012). Safety and dosing of bevacizumab (avastin) for the treatment of recurrent respiratory papillomatosis. Ann. Otol. Rhinol. Laryngol..

[9] Zeitels S.M., Barbu A.M., Landau-Zemer T., Lopez-Guerra G., Burns J.A., Friedman A.D., Freeman M.W., Halvorsen Y.D., Hillman R.E. (2011). Local injection of bevacizumab (Avastin) and angiolytic KTP laser treatment of recurrent respiratory papillomatosis of the vocal folds: A prospective study. Ann. Otol. Rhinol. Laryngol..

[10] Dheyauldeen S., Østertun Geirdal A., Osnes T., Vartdal L.S., Dollner R. (2012). Bevacizumab in hereditary hemorrhagic telangiectasia-associated epistaxis: Effectiveness of an injection protocol based on the vascular anatomy of the nose. Laryngoscope.

[11] Zagzoog F.H., Mogharbel A.M., Alqutub A., Bukhari M., Almohizea M.I. (2024). Intralesional cidofovir vs. bevacizumab for recurrent respiratory papillomatosis: A systematic review and indirect meta-analysis. Eur. Arch. Oto-Rhino-Laryngol..

[12] Hall S.R., Thiriveedi M., Yandrapalli U., Zhang N., Lott D.G. (2021). Sublesional Bevacizumab Injection for Recurrent Respiratory Papillomatosis: Evaluation of Utility in a Typical Clinical Practice. Ann. Otol. Rhinol. Laryngol..

[13] Pan Y., Lu Y., Huang H., Wang C., Han X., Hu H., Sun K., Li J., Zhang Y., Liu K. (2025). Quantifying Bevacizumab Efficacy in Recurrent Respiratory Papillomatosis. Laryngoscope.

[14] Derkay C., Malis D., Zalzal G., Wiatrak B., Kashima H., Coltrera M. (1998). A staging system for assessing severity of disease and response to therapy in recurrent respiratory papil·lomatosis. Laryngoscope.

[15] Martínez-Calvo J., Correa-Jiménez Ó., Alfaro-Murillo A. (2025). Bevacizumab in the Treatment of Recurrent Respiratory Papillomatosis: A Case of Isolated Nasal Involvement. Indian J. Otolaryngol. Head Neck Surg..

[16] Pähler Vor der Holte A., Fangk I., Glombitza S., Wilkens L., Welkoborsky H.J. (2020). Prognostic factors and risk factors for development and recurrence of sinonasal papillomas: Potential role of different HPV subtypes. Eur. Arch. Otorhinolaryngol..

[17] Pogoda L., Ziylan F., Smeeing D.P.J., Dikkers F.G., Rinkel R.N.P.M. (2022). Bevacizumab as treatment option for recurrent respiratory papillomatosis: A systematic review. Eur. Arch. Oto-Rhino-Laryngol..

[18] Walter H., Atfeh M. (2024). Evaluating the efficacy and safety of intralesional bevacizumab in the treatment of recurrent respiratory papillomatosis: A systematic review. Int. J. Pediatr. Otorhinolaryngol..

